# Machine Learning Versus Simple Clinical Models for Cochlear Implant Outcome Prediction

**DOI:** 10.3390/audiolres15060161

**Published:** 2025-11-21

**Authors:** Rieke Ollermann, Nils Strodthoff, Andreas Radeloff, Robert Böscke

**Affiliations:** 1 Division of Otolaryngology, Head and Neck Surgery, University of Oldenburg, 26129 Oldenburg, Germany; 2 Human Genetics, Faculty of Medicine and Health Science, University of Oldenburg, 26129 Oldenburg, Germany; 3 AI4Health Division, University of Oldenburg, 26129 Oldenburg, Germany; 4 Research Center Neurosensory Science, University of Oldenburg, 26129 Oldenburg, Germany; 5 Cluster of Excellence “Hearing4All”, University of Oldenburg, 26129 Oldenburg, Germany

**Keywords:** cochlear implant, hearing loss, prediction model, regression model, machine learning

## Abstract

**Background/Objectives:** Cochlear implantation is the most widely used treatment option for patients with severe to profound hearing loss. Despite being a relatively standardized surgical procedure, cochlear implant (CI) outcomes vary considerably among patients. Several studies have attempted to develop predictive models for CI outcomes but achieving accurate and generalizable predictions remains challenging. The present study aimed to evaluate whether simple and complex statistical and machine learning models could outperform the Null model based on various pre-CI implantation variables. **Methods:** We conducted a retrospective analysis of 236 ears with postlingual profound sensorineural hearing loss (SNHL) and measurable residual hearing (WRS_max_ > 0%) at the time of implantation. The median postoperative word recognition score with CI (WRS_65_(CI)) was 75% [Q1: 55%, Q3: 80%]. The dataset was divided using a 70:15:15 split into training (*n* = 165), validation (*n* = 35) and test (*n* = 36) cohorts. We evaluated multiple modeling approaches: different Generalized Linear Model (GLM) approaches, Elastic Net, XGBoost, Random Forest, ensemble methods, and a Null model baseline. **Results:** All models demonstrated similar predictive performance, with root mean squared errors ranging from 26.28 percentage points (pp) to 30.74 and mean absolute errors ranging from 20.62 pp to 23.75 pp. Coefficients of determination (R^2^) ranged from −0.468 to −0.073. Bland–Altman analyses revealed wide limits of agreement and consistent negative bias, while Passing–Bablok regression indicated calibration errors. Nonetheless, all models incorporating predictors significantly outperformed the Null model. **Conclusions:** Increasing model complexity yielded only marginal improvements in predictive accuracy compared with simpler statistical models. Pre-implantation clinical variables showed limited evidence of predictive validity for CI outcomes, although further research is needed.

## 1. Introduction

Hearing loss is one of the most common sensory impairments worldwide. For those with severe to profound sensorineural hearing loss (SNHL), cochlear implantation is the established standard of care in clinical practice today. A cochlear implant (CI) is an electronic hearing device that converts acoustic signals into patterned electrical stimulation of auditory nerve fibers, thereby bypassing the dysfunctional cochlear hair cells [1,2,3]. Despite incremental technological advancements in CI design, considerable variability in CI outcomes remains. Accurate prediction of postoperative CI performance would substantially improve clinical decision-making and enhance preoperative patient counselling regarding expected outcomes. Several studies have identified potential predictors of CI outcomes, such as age at hearing loss onset, duration of hearing loss, age at implantation, underlying etiology, CI experience, hearing aid use duration, CI manufacturer, percentage of active electrodes [4,5,6,7,8]. These predictors were used in various statistical models: regression models [4,5,6,7,9], random forests algorithms [10] and deep learning neural networks [11]. Comparative analyses between linear regression and machine learning models for CI outcome prediction showed modest advantages for ensemble-based approaches [12]. Reported model performance varied widely, explaining between 10% and 60% of outcome variance [4,6,8,13].

A previously reported general linear regression model (GLM) for the word recognition score with CI at 65 dB SPL (WRS_65_(CI)) suggested three predictors in a cohort of Cochlear™ recipients: Maximum word recognition score (WRS_max_), age at implantation and word recognition score with hearing aids at 65 dB SPL (WRS_65_(HA)). The GLM showed a median mean absolute error (MAE) of 13 percentage points (pp) for predictions of WRS_65_(CI) in a subgroup with 4FPTA ≤ 80 dB HL [9] and a median MAE of 11.5 pp for WRS_65_(CI) in a subgroup with preoperative WRS_max_ > 0% [14]. These variables have been consistently identified across multiple studies as putative predictors of postoperative cochlear implant outcomes [4,8,15,16,17].

The aim of this study was to evaluate whether advanced machine learning algorithms could enhance the prediction of CI word recognition outcomes beyond traditional statistical models.

## 2. Methods

This study was approved by the Medical Ethics Committee of the University of Oldenburg (application #2024-021).

### 2.1. Patient Cohort

This retrospective analysis included CI recipients treated at our center (ENT university clinic Oldenburg at Evangelisches Krankenhaus Oldenburg, Germany) between 2007 and 2023, receiving CIs from Cochlear (Cochlear Deutschland GmbH & Co. KG, Hannover, Germany), Med-EL (MED-EL Elektromedizinische Geräte Gesellschaft m.b.H., Innsbruck, Austria) or Advanced Bionics (Advanced Bionics LLC, Valencia, CA, USA). The study period was defined because earlier records were either incomplete or did not meet the inclusion criteria. The endpoint of 2023 was chosen to ensure that only fully completed follow-up years were included, as 12-month outcome measurements for CI recipients implanted in 2024 were not yet available at the time of data extraction in 2025.

Inclusion criteria were postlingual sensorineural hearing loss with onset at ≥6 years of age, preoperative residual hearing (WRS_max_ > 0%), and ≥12 months of CI rehabilitation.

Exclusion criteria comprised patients with etiologies documented to negatively impact cochlear implant outcomes (e.g., neuroinflammatory disorders, neurodegenerative conditions, and vestibular schwannoma) [4], individuals with intellectual disabilities that could compromise speech recognition testing reliability, and non-native German speakers whose linguistic background might confound word recognition assessments.

From an initial databank of 667 patients with 828 implanted ears, application of the inclusion and exclusion criteria yielded a final analytical cohort of 205 patients and 236 implanted CIs (174 (~85% unilaterally implanted), 31 (~15% bilaterally implanted).

An additional analysis was conducted on a subcohort consisting exclusively of Cochlear™ recipients, as previous studies have indicated that the predictive accuracy of word recognition outcomes tends to be slightly higher in a more homogenous subcohort [18].

### 2.2. Predictor Variables

Based on previous studies [4,5,6,7,8,16] and clinical relevance, seven predictor variables were selected:

- Age at onset of hearing loss (years);
- Duration of hearing loss (months);
- Age at implantation (years);
- Preoperative four-frequency pure tone average (4FPTA: 0.5 kHz, 1 kHz, 2 kHz, 4 kHz) of the implanted ear;
- Preoperative word recognition score at 65 dB with hearing aids (WRS_65_(HA)) of the implanted ear;
- Preoperative maximum word recognition score (WRS_max_) of the implanted ear;
- CI experience (defined as the time span between the implantation date and the WRS_65_(CI), with a minimum rehabilitation period of 12 months).

### 2.3. Data Preparation

A 70:15:15 split strategy (70% training, 15% validation, 15% test) was implemented. Hyperparameter optimization and cross-validation procedures were performed on the training set, with the validation set used for model comparison and the test set reserved for final evaluation.

Missing data were imputed using multiple imputation by chain equations (MICE) with m = 5 using the predictive mean matching (PMM) method. To prevent data leakage, imputations were first trained on training data. Subsequently, the trained MICE model was used to impute missing values in the validation and test sets via its transform functionality. This approach preserves the multivariate structure of the data and avoids the biases introduced by simpler methods like mean imputation.

### 2.4. Models

Null model

A null model is a baseline model that assumes the outcome variable can be predicted without using any predictors. It serves as a reference point for evaluating whether more complex models, which include predictors, offer better predictive performance. In our analyses, the null model predicts the median of the training data outcome (WRS_65_(CI)) for all observations, allowing us to assess whether incorporating additional variables yields statistically significant gains in model accuracy.

Generalized Linear Model (GLM)

We employed four different GLM-based modeling approaches.

GLM_Full employed linear regression with Gaussian distribution, incorporating all eight predictor variables.

GLM_Backward used the same linear regression framework but applied AIC-based backward stepwise elimination starting from the full model. At each step, the removal of each predictor was tested individually and the one with the greatest AIC improvement was dropped. This process was repeated iteratively until no further improvement in AIC could be achieved. This resulted in the equation: WRS_65_(CI) = 72.6377 − 0.1960 × age at implantation + 0.1962 × WRS_max_.

The GLM_External model implemented the published logistic regression model from Hoppe et al. [9], using the equation WRS_65_(CI) = 100/(1 + exp(−(β_0_ + β_1_ × WRS_max_ + β_2_ × Age + β_3_ × WRS_65_(HA)))) with fixed coefficients (β_0_ = 0.84, β_1_ = 0.012, β_2_ = −0.0094, β_3_ = 0.0059). This external model applies a logistic transformation to model the nonlinear relationship between predictors and WRS_65_(CI). In the original model, WRS_65_(CI) scores were converted into 20 binary (yes/no) responses [9].

Since the original model was validated in a cohort with residual hearing (WRS_max_ > 0%), we applied the same inclusion criterion throughout this study to ensure comparability.

GLM_External_Backward follows the same formulation as the GLM_External, but with re-estimated regression coefficients (β) based on the present study population (β_0_ = −2.257 × 10^1^, β_1_ = −9.056 × 10^−17^, β_2_ = 3.574 × 10^−16^, β_3_ = 4.073 × 10^−16^).

Generalized Additive Model (GAM)

GAM is more flexible than GLM for modelling complex, nonlinear data. Unlike GLMs with fixed coefficients, GAM uses smoothing splines to model relationships between response variables and predictors [19]. In our analysis, we applied smoothing splines to each predictor. The model is fitted with a gaussian family on the training data.

Elastic Net

Elastic net is a regularized linear regression method that combines L1 (Lasso) and L2 (Ridge) penalties to improve model performance. This technique balances feature selection (L1) and coefficient shrinkage (L2) to prevent overfitting and enhance generalizability, making it particularly useful when predictors are highly correlated [20]. We performed grid search with 10-fold cross-validation for hyperparameter optimization (α: 11-point grid from 0 to 1; λ: automatically selected). The final model was then trained on the full training set using the optimal α-λ combination.

Random Forest

Random Forest is an ensemble method that uses multiple decision trees to enhance model robustness and generalizability. It relies on bagging, where predictions are aggregated from decision trees trained on bootstrap samples [21,22]. This reduces overfitting by lowering tree variance. This approach can capture complex, nonlinear relationships and interactions among predictor variables, making it particularly suitable for predicting heterogeneous outcomes in CI recipients. In our analysis, we performed a grid search to tune hyperparameters, including the number of trees (between 100 and 1500), number of predictors randomly selected at each split (2 to 6), the minimum number of samples in nodes (1, 3, 5, 10), and the maximum of nodes per tree (between 0 and 200).

eXtreme Gradient Boosting (XGBoost)

XGBoost is an advanced ensemble learning technique based on the principles of boosting. Boosting combines multiple weak predictive models, typically decision trees, into a single strong model. Each model in the sequence corrects residual errors from the previous ones, improving prediction accuracy. Gradient boosting adjusts model parameters via gradient descent to minimize residual errors [23,24]. XGBoost has shown the potential to accurately predict clinical outcomes due to its iterative refinement, which enhances model precision, and its inherent robustness in managing missing or imbalanced data. This methodological strength renders it particularly well-suited for the analysis of complex, multifactorial outcomes in CI recipients. In our analysis, an enhanced grid search was performed to tune hyperparameters, including the learning rate (0.01, 0.05, 0.1, 0.2, 0.3), maximum tree depth (3 to 10), row subsampling (0.7 to 1.0), column subsampling per tree (0.7 to 1.0), and minimum child weight (1, 3, or 5). Models were trained up to 2000 boosting rounds with early stopping after 100 rounds without improvement on a held-out validation set.

Ensemble

Ensemble methods have demonstrated particular effectiveness in clinical prediction modeling, as they can enhance generalizability across diverse patient populations and clinical settings—an important consideration when modeling outcomes in cochlear implant recipients.

We implemented two ensemble methods combining predictions from the top three models based on validation set performance: Null model, Elastic Net and XGBoost. The simple ensemble calculated the arithmetic mean of the three model outputs to improve prediction stability and reduce variance. The weighted ensemble assigned each model’s contribution proportional to its inverse validation RMSE, favoring better-performing models in the final prediction.

### 2.5. Model Evaluation and Data Analysis

Data were collected in Microsoft Excel. All calculation, modelling, performance evaluation and figure generation were done using RStudio ([25], version 2024.12.1+563) with assistance from Claude ([26], version 4.0 (2024)). The outputs were reviewed and validated by the authors. Baseline characteristics of the cohort were analyzed using statistical approaches. Shapiro–Wilk normality testing was performed for each continuous variable. Variables with normal distribution were summarized using the mean and standard deviation (SD), whereas non-normally distributed data were described using the median and the percentiles at 25% to 75% (median ± Q1, Q3). Model performance was assessed using root mean squared error (RMSE), mean absolute error (MAE), and the coefficient of determination (R^2^) (calculated as 1—SS_residual/SS_total), with the latter representing traditional R^2^ for linear models and pseudo-R^2^ for nonlinear models. Models were primarily ranked by RMSE due to its direct clinical interpretability in word recognition scores (WRS). R^2^ and MAE are reported as supplementary performance indicators. Predicted scores were aligned with the corresponding observed values, and performance metrics were computed accordingly.

To assess the agreement between predicted and actual outcomes, Bland–Altman analysis was performed. Bias and limits of agreement (LoA) were calculated for each model to quantify systematic and individual-level error.

Passing-Bablok regression was employed to assess calibration performance. This non-parametric method provides estimates of proportional bias (slope) and constant bias (intercept), allowing for a comprehensive evaluation of systematic deviations from perfect predictions.

To determine whether model performances differed from the Null model, bootstrap resampling was used. Repeated iterations were performed to generate confidence intervals for RMSE, MAE and R^2^ differences.

## 3. Results

### 3.1. Patient Characteristics

A total of 236 implanted ears of adult patients with postlingual hearing loss and residual preoperative speech perception (WRS_max_ > 0%) were included. After applying a 70:15:15 split, the training cohort comprised 165 implanted ears. Table 1 summarizes the distribution of the cohort’s key clinical variables.

### 3.2. Model Performance Analysis

We assessed predictive performance across a range of models, including GLM_Full, GLM_Backward, GLM_External_Backward, GLM_External, GAM, Elastic Net, Random Forest, XGBoost, and two ensemble approaches (Weighted and Top3 models). Performances were benchmarked against the Null model.

**Figure 1:**
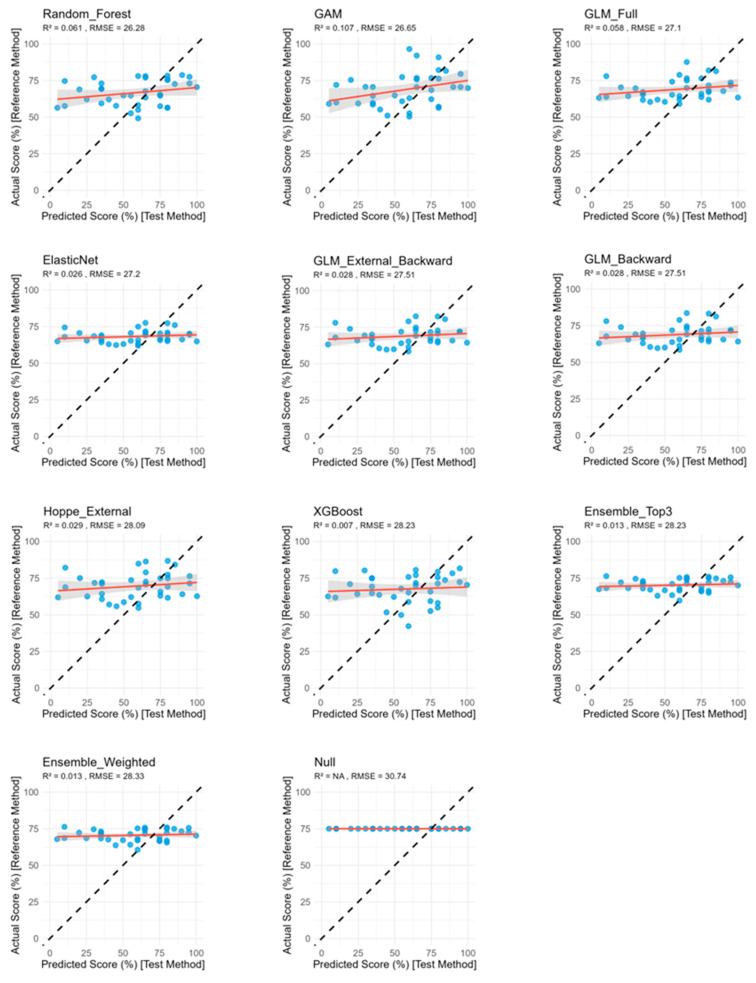
Actual versus predicted CI-outcome across different models using independent train/test splits. The black dashed line represents perfect prediction, and the red line shows the linear fit with confidence bands (grey). In all plots, the x-axis shows the predicted WRS_65_(CI) [%] and the y-axis shows the actual WRS_65_(CI) [%]. Blue dots represent the implanted ears in the test set cohort. RMSE = root mean squared error; GLM = generalized linear regression model; GAM = generalized additive model; XGBoost = eXtreme Gradient Boosting; *n* = 36.

All models demonstrated comparable RMSE and MAE, ranging between 26.28 pp and 30.74 percentage points (pp) and 20.62 pp and 23.75 pp, respectively (Table 2, Figure 1). R^2^ were consistently low and negative (−0.468 to −0.073), indicating poor model fit. Among the predictive models, Random Forest yielded the most favorable performance, with the lowest error metrics and highest R^2^ value. The different GLM models demonstrated marginally poorer accuracy, while the Null model performed worst, exhibiting the highest error metrics and lowest R^2^ (Table 2, Figure 1).

Across models, predicted outcome values spanned a broad range (Figure 1), reflecting the models’ ability to capture nearly the full spectrum of outcome variability.

**Figure 2:**
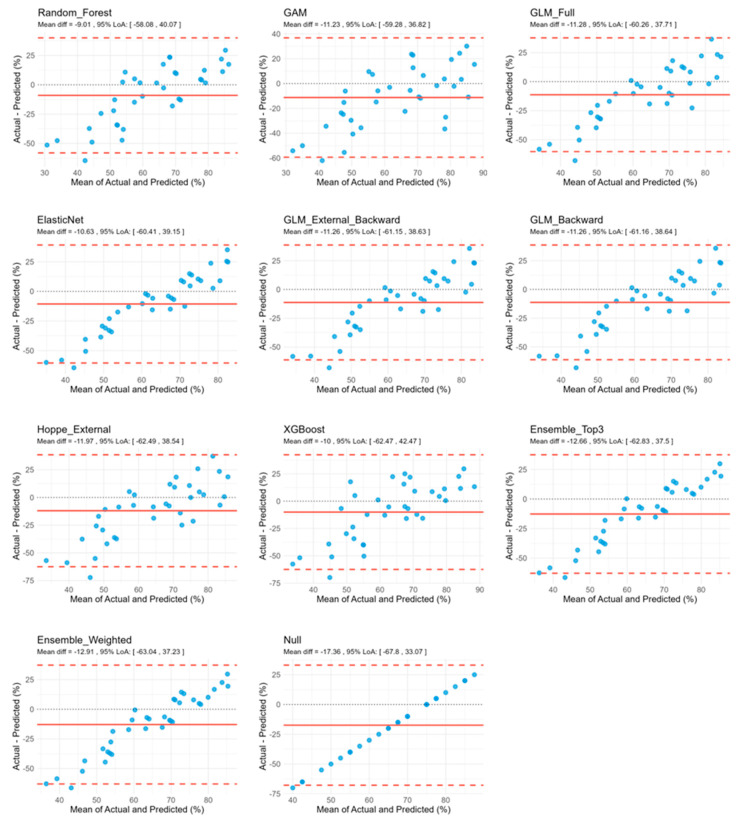
Bland–Altman analyses across different model performances. Mean difference and limits of agreement (LoA) were calculated for each model. Blue dots represent the implanted ears in the test set cohort. Dashed red lines represent the upper and lower LoA, while the red solid line shows the mean difference between actual and predicted values, indicating the average of bias of the model. The black dotted line represents the line of no difference (zero bias), indicating a perfect agreement between actual and predicted scores. *n* = 36.

Bland–Altman-analysis revealed consistently wide limits of agreement (LoA) for all models, ranging from ~96 to 105 pp, indicating substantial individual-level prediction error and limited agreement with actual outcomes. In addition, all models exhibited negative bias (range: −9.01 pp to −17.36 pp), suggesting a systematic tendency to underpredict outcomes (Table 2). Visual inspection of Bland–Altman plots further supported the presence of systematic bias. All models exhibited a diagonal pattern of residuals, rather than random scatter around zero, indicating systematic deviation from perfect prediction. The inclusion of predictors introduced additional dispersion along this diagonal trend, consistent with a modest but insufficient increase in explained variance (Figure 2).

These findings were corroborated by Passing-Bablok (Pb) regression, which identified notable calibration errors across models. The slopes were markedly less than 1 (range: 0.019–0.177), indicating a proportional underprediction, particularly at higher outcome values. Concurrently, the intercepts were consistently large and positive (range: 58.66 to 69.48 pp), reflecting a substantial systematic offset (Table 2).

Although overall predictive performance was limited, all models incorporating predictors significantly outperformed the Null model in terms of RMSE and R^2^. In terms of MAE, all models except GAM, Hoppe_External and XGBoost demonstrated statistically significant improvement (Table 3).

The improved performance metrics in all models over the Null model suggest that the inclusion of predictors contributes to enhanced predictive accuracy of the models, even if overall explanatory power remains limited.

Subgroup analyses were conducted in a subset of Cochlear™ recipients (*n* = 16 in the test cohort) to evaluate model performance in a more clinically homogenous population, which was previously shown to have a slightly better, although not significant, prediction accuracy in the Hoppe_External model [18]. Within this group, predictive accuracy was marginally improved across all models compared to the entire cohort (Table S1, Figure S1). Notably, Hoppe_External achieved the best performance, while XGBoost performed worst, exhibiting the highest error metrics and lowest R^2^ (Table S1, Figure S1). Similar to the entire cohort, predicted values remained confined to a narrow range, and correlations between actual and predicted CI outcomes were weak (Figure S1).

The Bland–Altman analysis showed wide LoAs (ranging from ~77 to ~90 pp) and consistently negative biases (−3.91 to −8.75 pp), again indicating a persistent tendency toward underprediction (Table S1). Residuals followed a diagonal distribution in Bland–Altman plots, and the inclusion of predictors contributed to increased dispersion along this trend (Figure S2).

Passing–Bablok regression results corroborated these patterns, with slopes substantially below 1 (range: 0.025–0.168), indicating persistent proportional underestimation at higher observed values. Intercepts remained large and positive (range: 59.01 to 70.88 pp), further highlighting systematic prediction error across all models (Table S1).

The improvement of the performance metrics over the Null model was not significant (Table S2), which might be due to the small sample size.

### 3.3. Feature Importance for CI-Outcome Predictions

**Figure 3:**
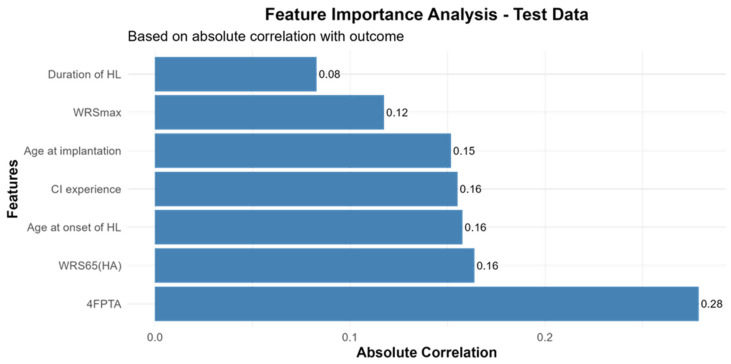
Feature importance analysis based on absolute correlation with CI outcome. The features WRS_max_, CI experience, age at implantation, WRS_65_(HA), age at onset of hearing loss (HL), duration of HL and the four-frequency pure tone average (4FPTA) were correlated to the WRS_65_(CI) to assess the importance of these variables. *n* = 36.

Given the limited predictive accuracy of all models, we examined feature importance by assessing correlations between predictor variables and WRS_65_(CI) using Pearson’s correlation coefficient. Across the entire cohort, all predictors exhibited weak to moderate associations with CI outcome, with preoperative 4FPTA demonstrating the strongest correlation. Notably, duration of hearing loss (HL) and WRS_max_ showed the weakest association with CI outcomes (Figure 3).

In the Cochlear™ recipient subgroup, age at implantation emerged as the strongest predictor, with a moderate correlation approaching an r ≈ 0.5. Conversely, WRS_65_(HA), 4FPTA and duration of hearing loss exhibited weak correlations with the CI outcome (Figure S3).

These findings suggest that the limited predictive performance of current models may stem from insufficiently informative predictors.

## 4. Discussion

In this study, we systematically compared the predictive performance of traditional regression- and more complex machine learning-based models against the Null model in predicting word recognition outcomes among CI recipients. Across all approaches, predictive performance was limited. The RMSE and MAE values were similar across models, with RMSE ranging from 26.28 pp to 30.74 pp and MAE from 20.62 pp to 23.75 pp, respectively. However, all predictive models significantly outperformed the Null model. These results are consistent with, albeit slightly higher than, error ranges reported in previous studies. Shafieibavani et al. [12] compared patient cohorts of three different centers (Vanderbilt University Medical Center (VUMC), Ear Science Institute Australia (ESIA), and Medizinische Hochschule Hannover (MHH)) by using an XGB-RF model and showed MAE ranges from 17.9 to 21.8 pp. Similarly, a recent study compared XGBoost with a traditional regression model and found comparable MAEs ranging between 17% and 22% [27], consistent with the findings from Shafieibavani et al. [12]. In addition, when outcomes were classified into quintile percentiles, XGBoost showed improved predictive performance over the traditional regression [27]. Crowson et al. [11] analyzed outcomes either as individual data points or categorized those into three groups: high, mid and low performers. They reported an RMSE of 25.3 for XGBoost and 0.57 for neural networks when only numerical variables were included. Notably, inclusion of text-based variables in the neural network model reduced classification accuracy from 95.4% to 73.3% and increased RMSE to approximately 25 pp [11], indicating decreased predictive performance with the addition of textual data. Another recent study employed a decision tree regression model and reported a MAE of 17–18 pp, with a relatively high standard deviation of ~12.5 to 14.5 pp [28], reflecting substantial variability in the model’s predictions.

Hoppe et al. [14] reported a lower MAE of 11.5 pp in a more homogeneous cohort of Cochlear™ recipients with WRS_max_ > 0%. Although we applied identical inclusion criteria in our subgroup analysis, predictive accuracy did not reach the same level. Several factors may explain the discrepancy. First, our cohort had a broader implantation timeframe (2007–2023) with heterogeneous cochlear implant generations. Second, our Cochlear™ subgroup had a smaller sample size. Third, institutional differences such as surgical techniques, audiological protocols, and patient selection criteria may have contributed to the performance gap between studies.

The overall model fit in our models was negligible, with R^2^ values ranging from −0.468 to −0.073. In all models, predictions exhibited a narrow range and deviated systematically from measured outcomes, as confirmed by Bland–Altman analyses and Passing–Bablok regression. While R^2^ is commonly interpreted as explained variance, this interpretation does not apply in non-linear models, where R^2^ functions more generally as an indicator of goodness of fit. Nevertheless, the broader literature reports highly variable explained variances ranging between 10% and 66% [4,5,8,10,11,12,29]. In these studies, higher R^2^ values in smaller cohorts may reflect statistical overfitting rather than superior predictive capability. While overfitting is detected through validation/test set performance, small test sets (such as our *n* = 16 subgroup) face the additional limitation of reduced representativeness. Smaller cohorts may not adequately capture population diversity, limiting the generalizability of performance estimates even when overfitting is not present. This should be considered when interpreting our subgroup analysis results. However, a previous study also suggested that sample size alone was unlikely to improve model performance [12]. Across the literature, a wide range of predictor variables have been employed, including age at implantation, duration of hearing loss, preoperative tone audiometry and speech recognition, duration of CI experience, the area ratio of ventral cochlear nerve to facial nerve at the cerebellopontine angle, and etiology [4,5,8,10,12,29,30]. Our findings indicate that the variables included in our models were insufficient to predict post-implantation outcomes for our cohort.

Feature importance analysis corroborated this interpretation: Classical predictors—including age at onset of hearing loss (years), duration of hearing loss (months), age at implantation (years), preoperative 4FPTA of the implanted ear, WRS_65_(HA) of the implanted ear, preoperative WRS_max_ of the implanted ear, CI experience (months)—exhibited only weak to moderate correlations with WRS_65_(CI). Our results are in contrast to previous studies that identified WRS_max_, WRS_65_(HA), and age at implantation as relevant predictors for CI outcome [4,8,15,16,17].

Notably, increasing model complexity—via ensemble methods, Random Forest, gradient boosting, or GAM—did not improve predictive accuracy compared with simpler regression approaches (GLMs with or without variable selection). This aligns with a prior study suggesting that prediction accuracy is constrained by the quality of the input variables, rather than by model choice [12].

This study has several important limitations that should be considered. The cohort exhibited substantial heterogeneity, encompassing a wide implantation timeframe (2007–2023), multiple CI manufacturers, and clinical methodologies that evolved over time. Such temporal and technical variability may have introduced noise into the analyses. As reported by Blamey et al. [4,30], the relative importance of predictive variables can change over time. Thus, future investigation stratifying by implantation period may yield additional insights. However, previous findings indicate that temporally defined subgroups may not differ substantially from randomly distributed subgroups [28]. Secondly, both ears of bilaterally implanted patients were analyzed as independent observations. Differences in the configuration of hearing loss—unilateral, bilateral, or asymmetric—could have affected model performance, as individuals often develop compensatory listening strategies that emphasize the better-hearing ear in daily communication. Thirdly, the models were limited to routinely collected data, primarily demographic and subjective audiometric variables. To enhance predictive accuracy, future work should integrate additional predictors such as imaging-derived metrics, genetic factors, cognitive measures, or more objective audiological assessments. Simply refining statistical or machine learning techniques without improving input variable quality is unlikely to yield substantial gains in predictive accuracy.

Finally, the statistical power was constrained by small test set sizes, potentially affecting the robustness of performance estimates. All models exhibited a systematic underprediction of outcomes. A nested cross-validation for evaluating the performance might lead to more reliable results. 

Subgroup analyses were conducted Further research, including external validation across diverse cohorts, will be needed. For this, public multi-center datasets with standardized test sets would be beneficial [31].

## 5. Conclusions

This study evaluated the predictive performance of linear and non-linear models for CI outcomes. Although the included variables have previously been identified as significant contributors, their assessment within more advanced modeling frameworks did not yield substantial improvements in predictive accuracy. Across all models, the error metrics were high and the explained variance remained minimal, underscoring the limited predictive value of currently available variables. Importantly, increasing model complexity did not enhance performance, suggesting that model architecture alone cannot compensate for weakly informative input features. These findings emphasize the need for the identification and integration of additional or alternative predictors—potentially including neuroimaging, genetic, cognitive, or more refined audiological measures—to improve the precision of CI outcome prediction. Moreover, rigorous external validation across diverse cohorts will be essential to strengthen both the robustness and generalizability of future predictive models.

## Figures and Tables

**Table 1:** Summary of patient characteristics.

Variable	N	Median [Q1, Q3]	Range
Duration of hearing loss (years)	222	17 [9–28]	1–80
Age at implantation (years)	236	63 [54–73]	18–88
CI experience (months)	223	12 [12–24.25]	10–140
Preoperative 4FPTA of the implanted ear (dB HL)	235	88.75 [80–96.25]	63.75–112.5
WRS_max_ (%)	236	30 [15–50]	5–100
WRS_65_(HA) (%)	236	15 [0–30]	0–60
WRS_65_(CI) (%)	236	75 [55–80]	5–100

Q1 = 25th percentile, Q3 = 75th percentile.

**Table 2:** Summary statistics of model performances.

Model	RMSE	MAE	R^2^	SD	Bias	Upper LoA	Lower LoA	Pb Slope	Pb Intercept	Correlation
Random_Forest	26.28	20.62	−0.073	25.04	−9.01	40.07	−58.08	0.094	61.25	0.248
GAM	26.66	21.20	−0.104	24.52	−11.23	36.82	−59.28	0.177	58.66	0.327
GLM_Full	27.10	21.26	−0.141	24.99	−11.28	37.71	−60.26	0.070	64.87	0.240
ElasticNet	27.20	21.23	−0.150	25.40	−10.63	39.15	−60.41	0.027	66.73	0.161
GLM_External_ Backward	27.51	21.19	−0.176	25.45	−11.26	38.63	−61.15	0.044	66.38	0.167
GLM_Backward	27.51	21.24	−0.176	25.46	−11.26	38.64	−61.16	0.046	66.27	0.168
Hoppe_External	28.09	21.50	−0.226	25.77	−11.97	38.55	−62.49	0.067	65.73	0.170
XGBoost	28.23	22.22	−0.238	26.77	−10.00	42.47	−62.47	0.036	65.54	0.082
Ensemble_Top3	28.23	21.97	−0.238	25.59	−12.66	37.50	−62.83	0.020	69.17	0.115
Ensemble_Weighted	28.33	22.05	−0.247	25.58	−12.91	37.23	−63.04	0.019	69.48	0.116
Null	30.74	23.75	−0.468	25.73	−17.36	33.07	−67.80	NaN	NaN	NA

RMSE = root mean squared error, MAE = mean absolute error, SD = Standard deviation; LoA = limits of agreement, Pb = Passing–Bablok; NaN = Not a Number; NA = Not available; Gray = baseline model, Green = best performing model; *n* = 36.

**Table 3:** Improvement in statistical metrics over the Null model.

Model	Mean RMSE Improvement [Lower CI, Upper CI]	*p*-Value	Mean MAE Improvement [Lower CI, Upper CI]	*p*-Value	Mean R2 Improvement [Lower CI, Upper CI]	*p*-Value
Random_Forest	4.446 [1.395, 7.577]	0.001	3.117 [−0.151, 6.416]	0.031	0.406 [0.122, 0.711]	0.001
GAM	4.063 [0.84, 7.199]	0.01	2.554 [−0.892, 6.17]	0.068	0.373 [0.084, 0.705]	0.01
GLM_Full	3.617 [1.012, 5.927]	0.003	2.454 [−0.39, 5.206]	0.039	0.339 [0.091, 0.624]	0.003
ElasticNet	3.51 [1.491, 5.342]	0.001	2.486 [0.154, 4.831]	0.02	0.331 [0.124, 0.583]	0.001
GLM_External_ Backward	3.221 [0.955, 5.35]	0.004	2.517 [0.022, 5.09]	0.023	0.305 [0.085, 0.571]	0.004
GLM_Backward	3.216 [0.872, 5.385]	0.004	2.468 [−0.045, 5.074]	0.032	0.304 [0.081, 0.575]	0.004
Hoppe_External	2.658 [−0.219, 5.369]	0.035	2.217 [−0.714, 5.078]	0.06	0.253 [−0.018, 0.55]	0.035
XGBoost	2.504 [−0.394, 5.315]	0.047	1.498 [−1.954, 4.855]	0.194	0.236 [−0.041, 0.51]	0.047
Ensemble_Top3	2.502 [0.941, 3.846]	0	1.761 [−0.289, 3.705]	0.043	0.239 [0.082, 0.42]	0
Ensemble_Weighted	2.404 [0.94, 3.669]	0	1.682 [−0.257, 3.532]	0.041	0.23 [0.082, 0.403]	0

CI = Confidence interval.

## Data Availability

The data presented in this study are available on request from the corresponding author. The data are not publicly available due to privacy restrictions.

## Supplementary Materials

The following supporting information can be downloaded at: https://www.mdpi.com/article/10.3390/audiolres15060161/s1, Figure S1: Actual versus predicted CI-outcome in Cochlear™ recipients across different models using independent train/test splits; Figure S2: Bland–Altman analyses across different model performances for the subcohort of Cochlear™ recipients; Figure S3: Feature importance analysis based on absolute correlation with CI outcome in Cochlear™ recipients; Table S1: Summary statistics of model performances in Cochlear™ recipients; Table S2: Improvement in statistical metrics over the Null model in Cochlear™ recipients.
