# Supplementary material for: Machine Learning Versus Simple Clinical Models for Cochlear Implant Outcome Prediction
Source: Audiol Res. 2025 Nov 21;15(6):161. doi: 10.3390/audiolres15060161 (PMC12641834; doi:10.3390/audiolres15060161)
Supplement: Supplementary file 1 [file audiolres-15-00161-s001.zip › audiolres-3957119-supplementary.pdf]

Supplements

***Table S1**: Summary statistics of model performances in Cochlear™ recipients*

**Model**	**RMSE**	**MAE**	**R^2**	SD	Bias	Upper LoA	Lower LoA	Pb slope	Pb intercept	correlation
Hoppe_External	19.40	15.45	0.009	19.63	-3.91	34.56	-42.38	0.168	59.01	0.287
ElasticNet	19.60	15.16	-0.012	19.60	-4.89	33.53	-43.31	0.054	67.60	0.227
GLM_Backward	19.93	15.71	-0.046	19.76	-5.59	33.15	-44.32	0.077	66.71	0.217
GLM_External_Backward	20.07	15.68	-0.061	19.95	-5.46	33.64	-44.55	0.096	65.35	0.215
Random_Forest	20.10	14.68	-0.064	20.29	-4.25	35.51	-44.01	0.065	66.21	0.157
GLM_Full	20.25	15.54	-0.080	19.97	-6.02	33.13	-45.17	0.126	63.90	0.236
Ensemble_Weighted	20.49	15.84	-0.106	20.15	-6.27	33.22	-45.72	0.025	70.88	0.106
Ensemble_Top3	20.51	15.82	-0.107	20.23	-6.07	33.58	-45.72	0.027	70.56	0.102
GAM	20.53	15.44	-0.110	20.07	-6.63	32.71	-45.96	0.127	64.47	0.230
Null	21.36	16.25	-0.202	20.13	-8.75	30.69	-48.19	NaN	NaN	NA
XGBoost	22.63	18.07	-0.349	22.89	-4.57	40.30	-49.44	0.037	68.40	0.041

RMSE = root mean squared error, MAE = mean absolute error. Gray = baseline model, Green: Best performing model; *n*=16.

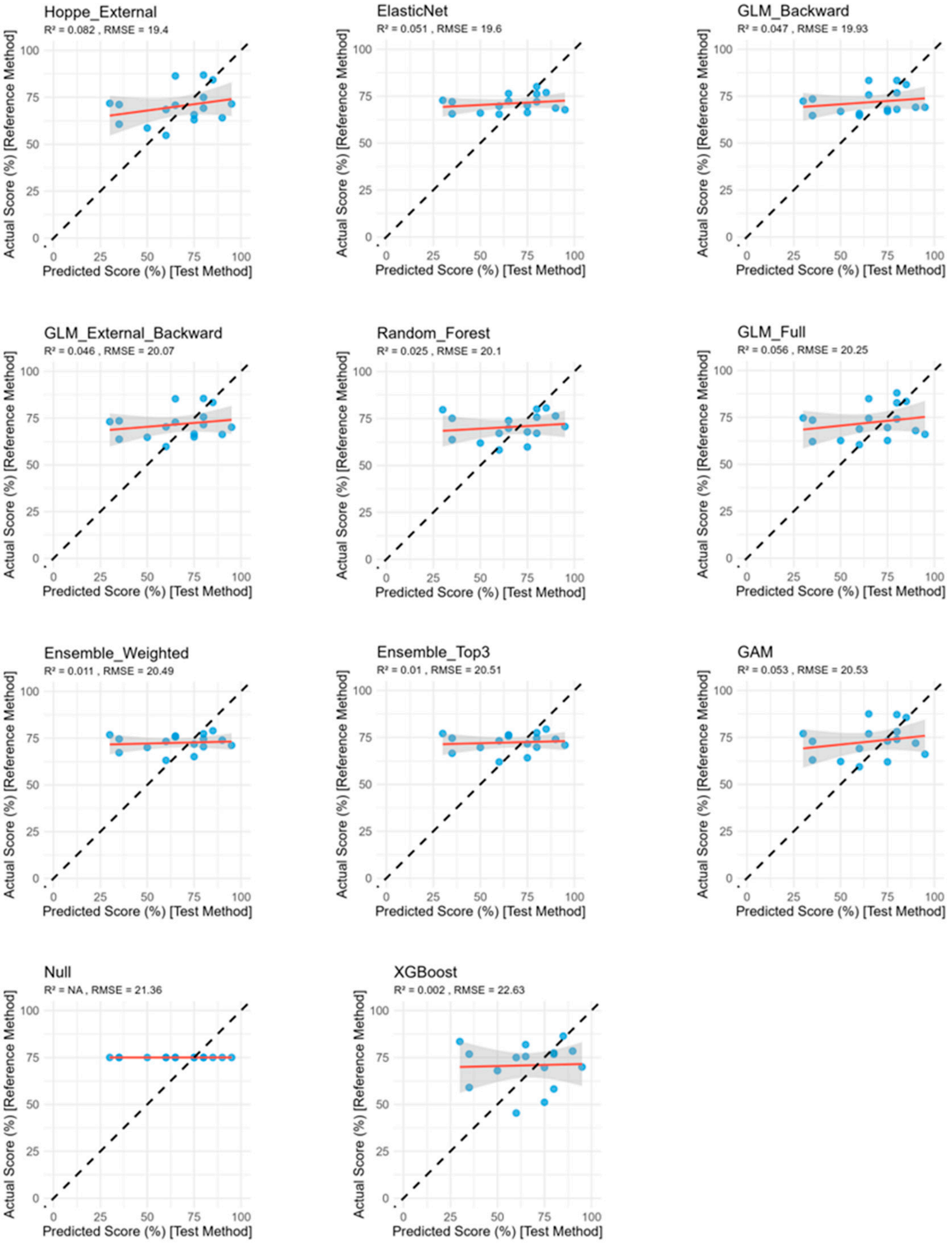

Figure S1: *Actual versus predicted CI-outcome in Cochlear™ recipients across different models using independent train/test splits. The black dashed line represents perfect prediction, and the red line shows the linear fit with confidence bands (grey). In all plots, the x-axis shows the predicted* WRS_{65}*(CI) [%] and the y-axis shows the actual* WRS_{65}*(CI) [%]. Blue dots represent the implanted ears in the test set cohort. RMSE = root mean squared error; GLM = generalized linear regression model; GAM =generalized additive model; XGBoost = eXtreme Gradient Boosting; n=16.*

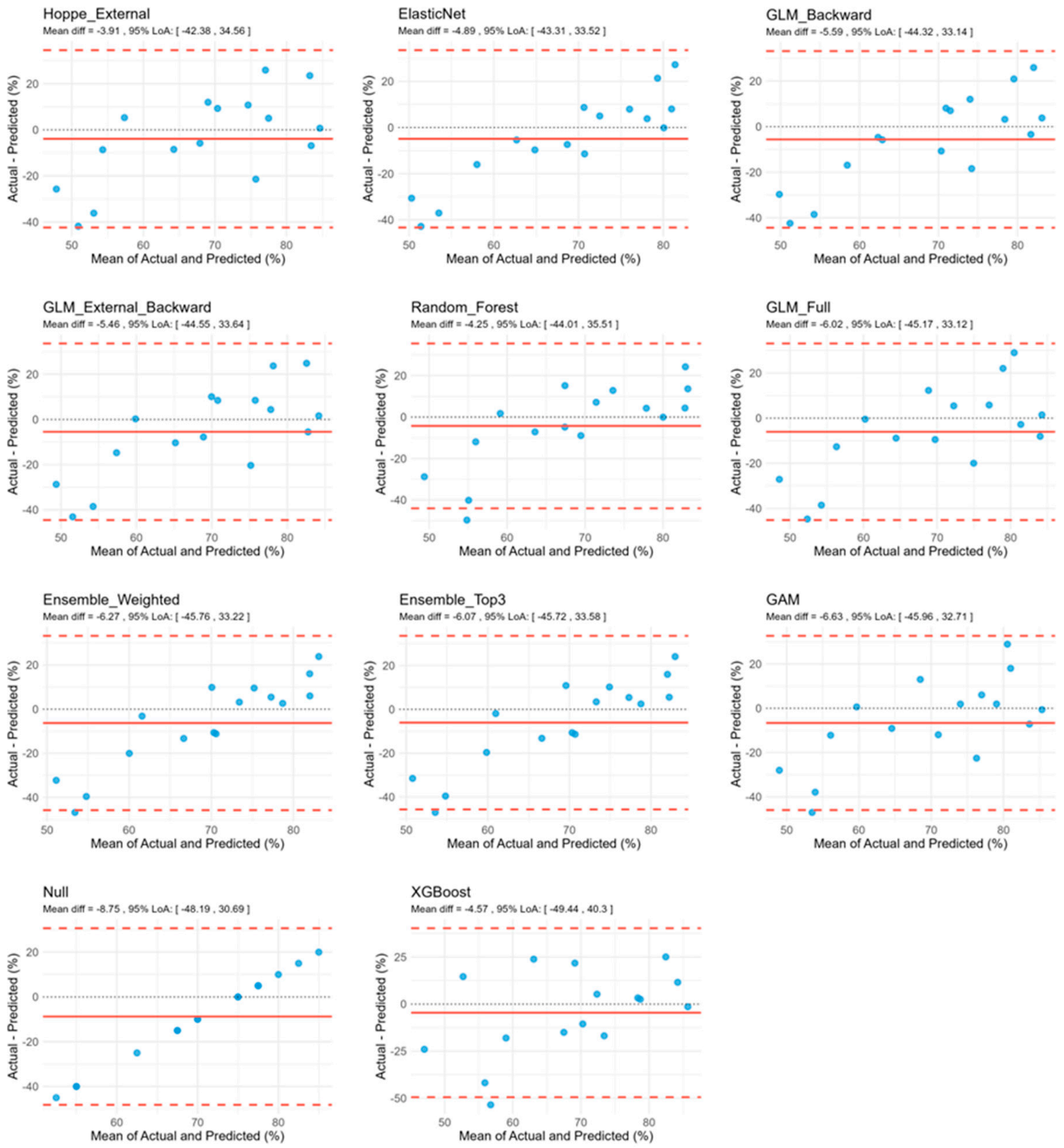

***Figure S2**: Bland-Altman analyses across different model performances for the subcohort of Cochlear™ recipients. Mean difference and limits of agreement (LoA) were calculated for each model. Blue dots represent the implanted ears in the test set cohort. Dashed red lines represent the upper and lower LoA, while the red solid line shows the mean difference between actual and predicted values, indicating the average of bias of the model. The black dotted line represents the line of no difference (zero bias), indicating a perfect agreement between actual and predicted scores. n= 16.*

***Table S2**: Improvement in statistical metrics over the Null model in Cochlear™ recipients*

Model	Mean RMSE improvement [lower CI, upper CI]	P-value	Mean MAE improvement [lower CI, upper CI]	P-value	Mean R^2 improvement [lower CI, upper CI]	P-value
Hoppe_External	1.877 [-1.843, 5.38]	0.159	0.777 [-3.419, 4.898]	0.369	0.211 [-0.266, 0.649]	0.159

ElasticNet	1.744 [-0.995, 4.031]	0.096	1.094 [-1.755, 3.686]	0.215	0.207 [-0.12, 0.551]	0.096
GLM_Backward	1.392 [-1.493, 3.921]	0.149	0.547 [-2.676, 3.677]	0.372	0.163 [-0.196, 0.496]	0.149
Random_Forest	1.391 [-1.735, 4.935]	0.242	1.606 [-2.515, 5.28]	0.217	0.165 [-0.206, 0.603]	0.242
GLM_External_Backward	1.231 [-1.715, 4.076]	0.205	0.534 [-3.01, 4.209]	0.393	0.142 [-0.247, 0.502]	0.205
GLM_Full	1.08 [-2.526, 4.402]	0.274	0.661 [-3.274, 4.548]	0.388	0.126 [-0.333, 0.537]	0.274
Ensemble_Weighted	0.883 [-1.043, 2.958]	0.214	0.403 [-1.872, 2.717]	0.372	0.108 [-0.119, 0.375]	0.214
Ensemble_Top3	0.871 [-1.249, 3.142]	0.245	0.42 [-2.091, 2.942]	0.382	0.106[-0.138, 0.393]	0.245
GAM	0.823 [-2.801, 4.372]	0.333	0.747 [-3.12, 4.74]	0.354	0.095 [-0.357, 0.518]	0.333
XGBoost	-1.221 [-5.2, 3.605]	0.717	-1.798 [-6.53, 2.664]	0.777	-0.17 [-0.767, 0.394]	0.717

CI = Confidence intervals; *n*=16.

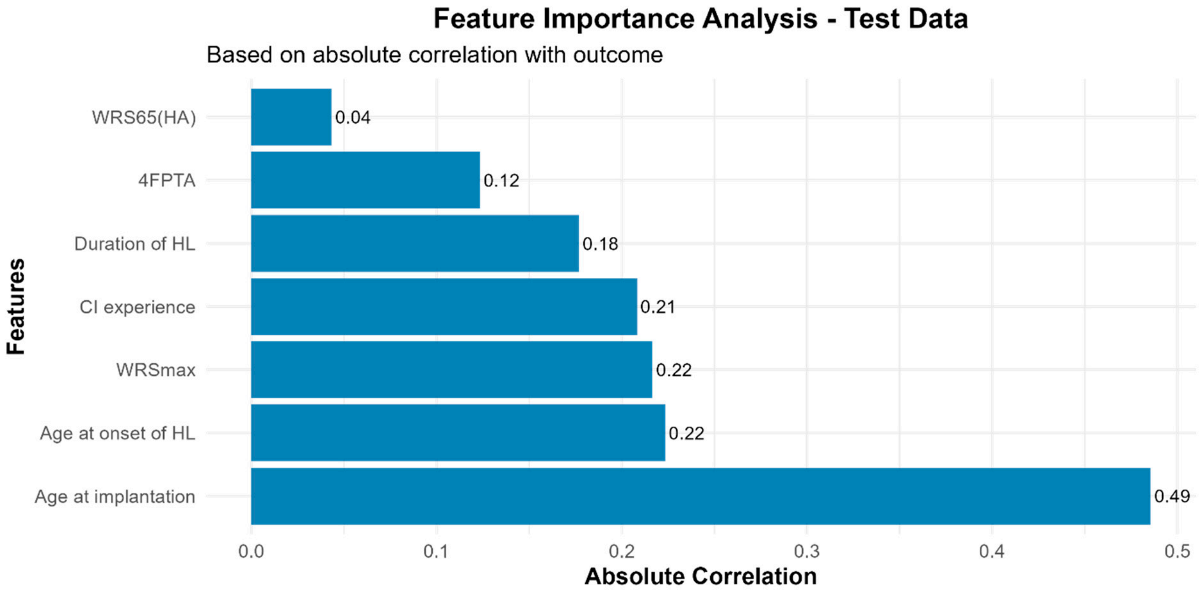

***Figure S3**: Feature importance analysis based on absolute correlation with CI outcome in Cochlear™ recipients. The features WRS_{65}(HA), the four-frequency pure tone average (4FPTA), CI experience, duration of hearing loss (HL), WRS_{max}, age at onset of HL, and age at implantation were correlated to the WRS_{65}(CI) to assess the importance of these variables. n=16.*
